# Male mice with large inversions or deletions of X-chromosome palindrome arms are fertile and express their associated genes during post-meiosis

**DOI:** 10.1038/s41598-018-27360-x

**Published:** 2018-06-12

**Authors:** Alyssa N. Kruger, Quinn Ellison, Michele A. Brogley, Emma R. Gerlinger, Jacob L. Mueller

**Affiliations:** Department of Human Genetics, University of Michigan Medical School, Ann Arbor, MI 48109 USA

## Abstract

Large (>10 kb) palindromic sequences are enriched on mammalian sex chromosomes. In mice, these palindromes harbor gene families (≥2 gene copies) expressed exclusively in post-meiotic testicular germ cells, a time when most single-copy sex-linked genes are transcriptionally repressed. This observation led to the hypothesis that palindromic structures or having ≥2 gene copies enable post-meiotic gene expression. We tested these hypotheses by using CRISPR to precisely engineer large (10’s of kb) inversions and deletions of X-chromosome palindrome arms for two regions that carry the mouse *4930567H17Rik* and *Mageb5* palindrome gene families. We found that *4930567H17Rik* and *Mageb5* gene expression is unaffected in mice carrying palindrome arm inversions and halved in mice carrying palindrome arm deletions. We assessed whether palindrome-associated genes were sensitive to reduced expression in mice carrying palindrome arm deletions. Male mice carrying palindrome arm deletions are fertile and show no defects in post-meiotic spermatogenesis. Together, these findings suggest palindromic structures on the sex chromosomes are not necessary for their associated genes to evade post-meiotic transcriptional repression and that these genes are not sensitive to reduced expression levels. Large sex chromosome palindromes may be important for other reasons, such as promoting gene conversion between palindrome arms.

## Introduction

In humans and mice, the sex chromosomes are enriched for large (>10 kb), nearly identical (>99% nucleotide identity) segmental duplications in palindromic orientation^1–4^. In mice, genes within large X-chromosome palindromes are expressed predominantly or exclusively in post-meiotic testicular germ cells^3^. This specific expression pattern is surprising, because most single-copy X-linked genes are transcriptionally repressed post-meiosis^5–8^. The mechanism by which palindrome-associated genes escape transcriptional repression is unknown. Two hypotheses have been suggested to explain this distinct expression pattern. First, palindromes may form secondary structures (e.g. palindrome arms pairing to form a hairpin) enabling their associated genes to evade transcriptional repression^3^. Intrachromosomal synapsis of palindrome arm pairing could facilitate the evasion of post-meiotic gene repression, which itself is a consequence of asynapsis-triggered meiotic sex chromosome inactivation^9–11^. Second, X-palindromic genes may be sensitive to reduced expression levels and thus require ≥2 gene copies^3^. Consistent with this, the mouse X chromosome also carries non-palindromic multicopy genes that are expressed specifically in post-meiotic cells^3^. To test the two hypotheses, individual palindrome arms must be genetically manipulated, *in vivo*.

To rigorously test whether palindrome structure or gene copy number are required for post-meiotic expression, we genetically dissected two mouse X-palindromes. We utilized CRISPR to generate large-scale (10’s of kb) inversions and deletions in mice of two X-palindrome arms harboring the *4930567H17Rik* and *Mageb5* (*Melanoma antigen gene family member b5*) gene families. We chose these two X-palindromes because they possess the canonical features of palindromes across mammals; they have >99% percent nucleotide identity between the two arms, the arms are >10 kb in length, and they harbor a gene family expressed predominantly in post-meiotic testicular germ cells. We also selected these two gene families because they have nucleotide variants that differ between the two gene copies, enabling detection of palindrome arm-specific expression of each gene copy. We found that for the *4930567H17Rik* and *Mageb5* palindromic gene families, palindrome structure is not necessary for regulating their associated post-meiotic gene expression, since mice containing palindrome arm inversions exhibit wild-type expression levels. We observed that deletion of a single palindrome arm, for both the *4930567H17Rik* and *Mageb5* gene families, reduces gene expression levels by half. Reduced expression levels did not lead to male infertility or spermatogenic defects in either case. This suggests that palindromes enrichment on the sex chromosomes is important for other reasons and that there are alternative, unknown mechanisms for palindrome-associated genes to evade post-meiotic repression.

## Results

### The mouse X chromosome harbors eight singleton palindromes

**Figure 1:**
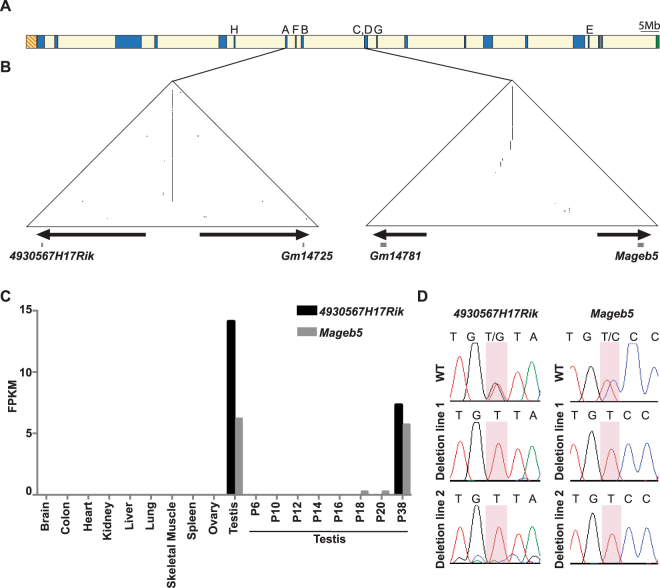
Singleton palindromes on the mouse X chromosome. (**A**) The location of the eight singleton palindromic regions on the mouse X chromosome are labeled by arm size, the sequence features of which can be found in Table 1. (**B**) Self-symmetry triangular dot plots of the two singleton X-palindromes to be studied, carrying the *4930567H17Rik* and *Mageb5* gene families, respectively. Each dot plot represents the palindromic X chromosome sequence (*4930567H17Rik* = chrX:70385921–70553920 and *Mageb5* = chrX:91624421–91790420) plotted against itself with a sliding window of 100 nucleotides (step size = 1 nucleotide). When the window of 100 nucleotides is identical to the sequence it is compared to, a dot is plotted. Segmental duplications in an inverted orientation are visualized as vertical lines. A visual representation of the palindrome arms (arrows) and the gene copies (squares) are plotted at the base of the triangular plots across the 168 kb *4930567H17Rik* and 166 kb *Mageb5* palindromic regions. (**C**) Expression levels of *4930567H17Rik* and *Mageb5* genes in adult tissues and juvenile testis, shown as FPKMs (number of fragments per kilobase per million mapped fragments). **D**) Sanger sequencing of RT-PCR products displaying the nucleotide differences that distinguish the two palindromic gene copies. In *4930567H17Rik*^*DelArm/Y*^ and *Mageb5*^*DelArm/Y*^ mice, expression is detected only from the remaining copy. WT = wild-type.

Large palindromes on mammalian sex chromosomes are typically found as isolated pairs of palindrome arms (singleton palindromes) or in complex arrays of palindromes. We investigated singleton palindromes, because they are more commonly found across mammalian sex chromosomes and can be genetically manipulated *in vivo* more precisely. Of the eight singleton palindromes on the mouse X chromosome (Table 1 and Fig. 1A), we selected two harboring the *4930567H17Rik* and *Mageb5* gene families, because they share canonical features of sex chromosome palindromes: >10 kb, >99% nucleotide identity between palindrome arms, harbor genes expressed specifically in testicular germ cells, and have a spacer sequence between the palindrome arms (Table 1 and Fig. 1B). Additionally, the palindrome carrying the *4930567H17Rik* gene family has the longest palindrome arm (65 kb), for a singleton palindrome, which will serve as a proof of principle for the manipulation of shorter palindrome arms.

**Table 1:** Sequence features of mouse X chromosome singleton palindromes.

Palindrome	Genes*	Arm Size (kb)^+^	Spacer size (kb)	Percent Identity^+^
A	*4930567H17Rik*	64.7	29.1	99.26
B	*Gm5640*	47.1	11.7	99.61
C	*Gm5071*	34.6	44.9	99.88
D	*Mageb5*	30.0	98.8	99.26
E	*3010001F23Rik*	28.5	47.8	99.49
F	*Xlr5a*	27.0	3.7	99.59
G	*Zxda*	21.4	67.8	99.56
H	*Gm773*	13.3	25.7	99.36

*In cases where two gene family members have different names, only one was selected (e.g. *Mageb5* was selected for gene family that has *Mageb5* and *Gm14781*). ^+^Palindrome arm size and percent identities between arms were identified from the “Segmental Dups” track of the UCSC genome browser mm10 mouse genome assembly.

We validated the exclusive expression of the *4930567H17Rik* and *Mageb5* gene families in post-meiotic round spermatids by reanalyzing previously published RNA-seq datasets from a tissue panel, juvenile testis (Fig. 1C) and sorted testicular germ cells (Supplementary Fig. 1). To determine if both gene copies of *4930567H17Rik* and *Mageb5* are expressed, we utilized individual nucleotide differences between gene copies. Sequencing of RT-PCR products for both *4930567H17Rik* and *Mageb5* show that both gene copies are expressed (Fig. 1D). Having confirmed that both gene copies are expressed exclusively in post-meiotic round spermatids, we proceeded to delete or invert individual palindrome arms to assess the importance of palindrome structure and gene copy number.

### Generation of mice carrying precise inversions and deletions of individual X-palindrome arms via CRISPR

**Figure 2:**
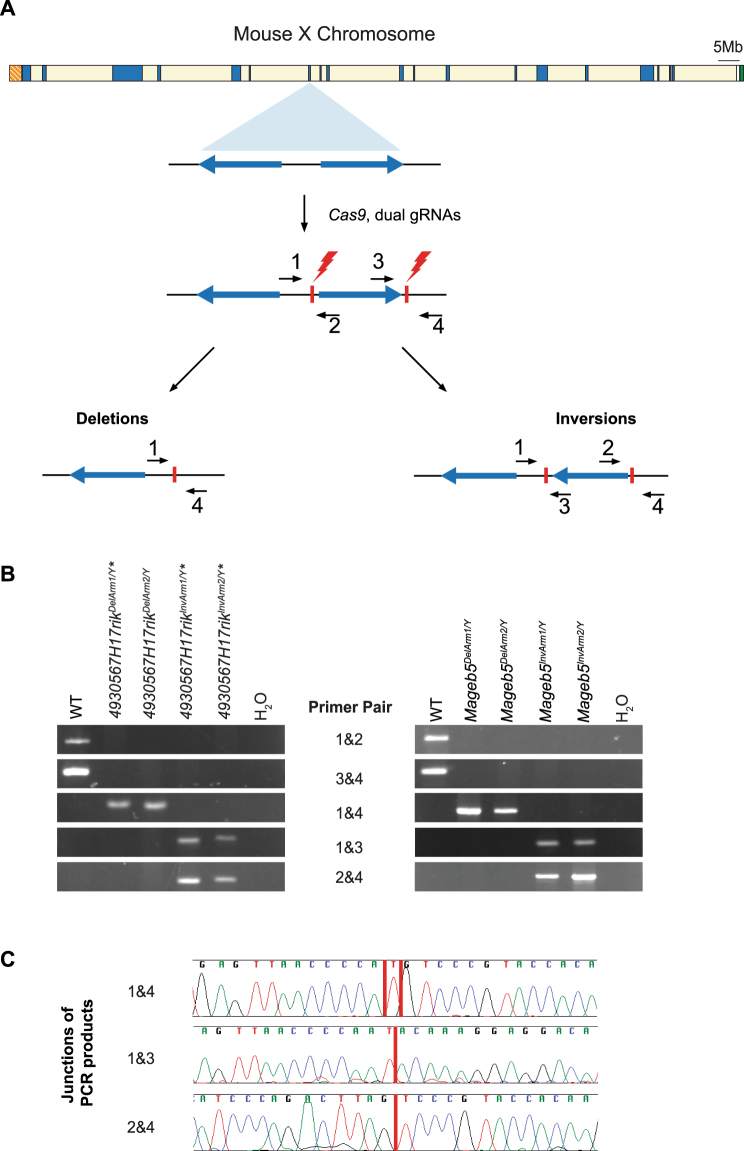
CRISPR strategy to generate large inversions and deletions of individual palindrome arms. (**A**) Schematic of the mouse X chromosome with a diagram of a singleton palindrome shown below. Palindrome arms are shown as blue arrows, sgRNA sites as red vertical lines and primers as black arrows. (**B**) PCR genotyping of DNA from the two independent mouse lines for each deletion and inversion of *4930567H17Rik* and *Mageb5* palindrome arms. Numbered primers from panel A used to amplify deletion (1&4) and inversion (1&3, 2&4) junctions (Supplementary Table 1). Full-length agarose gels are in Supplementary Fig. 4. WT = wild-type. (**C**) Representative example of Sanger sequencing chromatograms of the deletion and inversion junctions for *4930567H17Rik* palindrome arm rearrangements. Junction sites are shown with a vertical red line. The mm10 coordinates for the sequence removed in the *4930567H17Rik*^*DelArm1/Y*^ line is ChrX:70389542–70457358 and coordinates for the sequence inverted in the *4930567H17RiK*^*InvArm1/Y*^ are ChrX:70389544–70457357.

We utilized CRISPR/Cas9 technology to precisely invert or delete large X-palindrome arms in mice. Pronuclear injection of mouse zygotes with dual single guide RNAs (sgRNAs), targeting unique flanking regions of each palindrome arm, and use of a single stranded oligonucleotide donor enabled us to generate large (29 kb and 65 kb) inversions and deletions of individual arms for the *4930567H17Rik* and *Mageb5* X-palindromes (Fig. 2A). The single stranded oligonucleotide donors were used to promote inversions and deletions. We screened founder mouse lines with combinations of primers flanking the sgRNA cut sites in order to detect and distinguish inversions and deletions (Fig. 2A,B). We validated inversion and deletion junctions by PCR followed by Sanger sequencing (Fig. 2C). After ~300 pronuclear injections, we obtained 2 and 3 independent mouse lines carrying deletions of a single palindrome arm for the *4930567H17Rik* and *Mageb5* gene families, respectively. Similarly, after ~300 pronuclear injections we obtained 4 and 2 independent mouse lines carrying inversions of a single palindrome arm for the *4930567H17Rik* and *Mageb5* gene families, respectively. The independently obtained inversion and deletion junctions differed from each other by only a few nucleotides at the junctions. We also confirmed the deletions via Sanger sequencing of RT-PCR products to show that only a single sequence family variant was expressed in each mouse line (Fig. 1D). Eight independent mouse lines, backcrossed onto a C57BL/6J genetic background, were selected for further study, two lines carrying deletions and two lines carrying inversions for each of the two X-palindromic regions. Throughout this study, the genotypes for the eight deletion and inversion carrying lines are designated *4930567H17RiK*^*InvArm*^, *4930567H17Rik*^*DelArm*^, *Mageb5*^*InvArm*^, and *Mageb5*^*DelArm*^, followed by a ‘1’ or ‘2’ to denote individual mouse lines. Our dual sgRNAs combined with single stranded oligonucleotides approach was successful in generating 29 kb and 65 kb inversions and deletions of single palindrome arms.

### Disruption of palindrome structure, via inverting a single palindrome arm, does not affect the gene expression levels of the palindrome associated gene family

**Figure 3:**
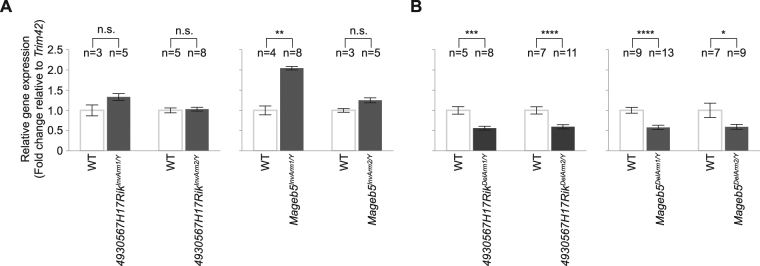
Male mice carrying *4930567H17Rik* and *Mageb5* palindrome arm inversions or deletions express their associated genes at wild-type and half of wild-type levels, respectively. (**A**) *4930567H17Rik* and *Mageb5* gene expression levels from testes of male mice carrying palindrome arm inversions as compared to wild-type controls. (**B**) *4930567H17Rik* and *Mageb5* gene expression levels from testes of male mice carrying palindrome arm deletions as compared to wild-type controls. For A and B, the gene expression values are shown as fold changes normalized to wild-type (WT = 1) and to the standard reference gene *Trim42* for two independent mouse lines, per genotype (inversion or deletion of *Mageb5* or *4930567H17Rik* palindrome arms). Error bars represent the standard error of the mean across the biological replicates. The number of biological replicates (n) examined per line is shown above each bar graph. Unpaired two-tailed t-tests, with a Welch correction, were performed to compare mean gene expression (n.s. = not significant, *P < 0.05, **P < 0.01, ***P < 0.001, ****P < 0.0001).

With the generation of mice carrying precise inversions of two different X-palindrome arms that disrupt palindrome structure, we tested whether palindrome structure is necessary for post-meiotic gene expression. If palindrome structure is necessary for post-meiotic gene expression, then we expected to abolish gene expression of *4930567H17Rik* and *Mageb5* in *4930567H17RiK*^*InvArm/Y*^ and *Mageb5*^*InvArm/Y*^ mice, respectively. We compared the testis gene expression levels of *4930567H17Rik* and *Mageb5* gene families via quantitative RT-PCR (RT-qPCR). Expression was normalized to *Trim42* and compared to wild-type controls. *4930567H17RiK*^*InvArm/Y*^ and *Mageb5*^*InvArm/Y*^ mice, in two independent mouse lines, express their associated genes at levels similar to wild-type mice (Fig. 3A). Consistent with their similar gene expression levels, *4930567H17RiK*^*InvArm/Y*^ and *Mageb5*^*InvArm/Y*^ mice are fertile and do no exhibit detectable spermatogenic defects. Assuming the post-meiotic-specific gene expression of *4930567H17Rik* and *Mageb5* is maintained in mice carrying palindrome arm inversions, our data demonstrate that palindrome structure is not required for gene expression of *4930567H17Rik* and *Mageb5*.

### Deleting a single palindrome arm reduces the gene expression level of the palindrome-associated gene family by half

Using mice carrying single palindrome arm deletions, we reduced the gene copy number of the palindrome-associated *4930567H17Rik* and *Mageb5* genes by half (from 2 gene copies to 1). We expected *4930567H17Rik* and *Mageb5* gene expression levels would be reduced by half in *4930567H17Rik*^*DelArm/Y*^ and *Mageb5*^*DelArm/Y*^ mice. We compared the testis gene expression levels of *4930567H17Rik* and *Mageb5* gene families via quantitative RT-PCR (RT-qPCR). Expression was normalized to *Trim42* and compared to wild-type controls. We found that *4930567H17Rik*^*DelArm/Y*^ and *Mageb5*^*DelArm/Y*^ mice, in two independent mouse lines, expressed their associated genes at approximately half the levels of wild-type males (Fig. 3B). The reduction of gene expression by half, in mice carrying palindrome arm deletions, is consistent with reducing gene copy number from 2 to 1.

### Male mice carrying deletions of individual palindrome arms do not exhibit defects in fecundity, testis histology, or number of round spermatids

**Figure 4:**
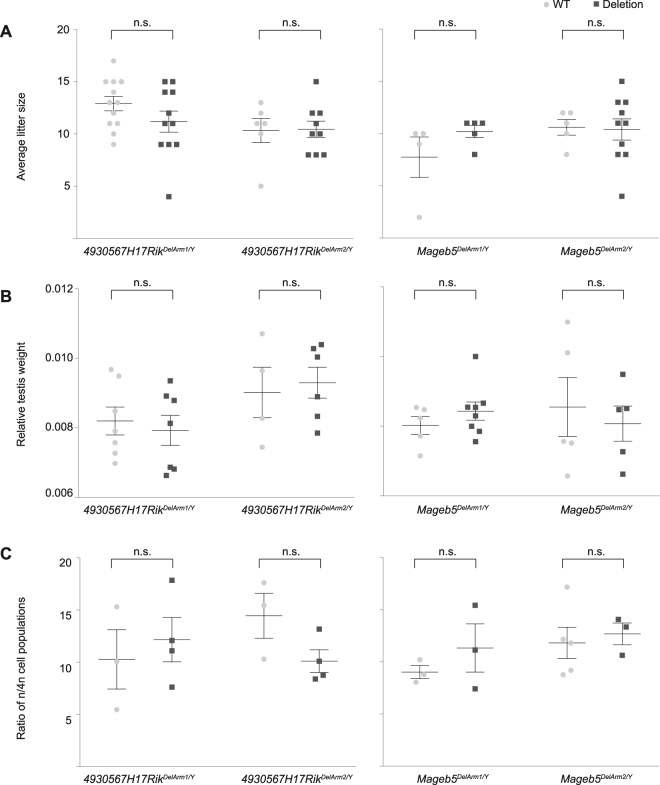
Male mice carrying *4930567H17Rik* and *Mageb5* palindrome arm deletions are fertile and do not display defects in spermatogenic cell population frequencies. (**A**) Multiple males from each *4930567H17Rik*^*DelArm/Y*^ and *Mageb5*^*DelArm/Y*^ line and control males (wild-type littermates) were mated to multiple CD1 females to assess fertility and fecundity. (**B**) Total testis weight (g) from *4930567H17Rik*^*DelArm/Y*^ and *Mageb5*^*DelArm/Y*^ lines were normalized to total body weight (g). (**C**) The frequency of post-meiotic round spermatids was assessed by normalizing the percentage of round spermatids (1n) to spermatocytes (4n). The spermatocyte population serves as a control and should be unaffected, because they do not express *4930567H17Rik* and *Mageb5*. Error bars represent the standard error of the mean of biological replicates. Statistical significance was determined using an unpaired T-test assuming the same standard deviation between populations and correction for multiple comparisons using Holm-Sidak correction.

The reduction of gene expression by half allows us to test whether male mice are sensitive to reduced gene expression of the *4930567H17Rik* and *Mageb5* gene families. We performed a systematic characterization of fecundity and post-meiotic spermatogenic development of *4930567H17Rik*^*DelArm/Y*^ and *Mageb5*^*DelArm/Y*^ mice. We found that *4930567H17Rik*^*DelArm/Y*^ and *Mageb5*^*DelArm/Y*^ mice are fertile and produce litter sizes and relative testis weights similar to wild-type controls (Fig. 4A,B). To detect potential defects in post-meiotic spermatid development, we examined histological sections of testis from *4930567H17Rik*^*DelArm/Y*^ and *Mageb5*^*DelArm/Y*^ mice. We did not observe defects in spermatid morphology, formation of the acrosome, or spermatid elongation (Supplementary Fig. 2). To assess whether the number of round spermatids were affected in *4930567H17Rik*^*DelArm/Y*^ and *Mageb5*^*DelArm/Y*^ mice, we quantified the number of round spermatids per testis as the ratio of round spermatids/spermatocytes (control) via FACs (Supplementary Fig. 3). The ratio of round spermatids/spermatocytes of *4930567H17Rik*^*DelArm/Y*^ and *Mageb5*^*DelArm/Y*^ mice was similar to wild-type males (Fig. 4C). Altogether, *4930567H17Rik*^*DelArm/Y*^ and *Mageb5*^*DelArm/Y*^ mice do not exhibit detectable defects in fecundity or post-meiotic spermatid development.

## Discussion

Our findings suggest that palindrome structure is not necessary for regulating post-meiotic gene expression levels for the *4930567H17Rik* and *Mageb5* gene families. Our findings also show that a reduced gene expression level of the *4930567H17Rik* and *Mageb5* gene families, via deletion of a single palindrome arm, does not result in spermatogenic defects or male infertility. It will be important to compare our studies with complete knockouts of the *4930567H17Rik* and *Mageb5* gene families. If either gene family is necessary for spermatogenesis, then it would support our findings that palindrome structure or copy number are not necessary for spermatogenesis.

Our findings suggest there are alternative mechanisms for X-palindromic genes to be expressed on the otherwise transcriptionally repressed X chromosome. A small number of X-linked single-copy genes are expressed in round spermatids^6,12^, indicating that multiple gene copies are not a strict requirement for post-meiotic gene expression from the sex chromosomes. Specific enhancers and transcription factors may have evolved to overcome the transcriptional repression associated with post-meiotic sex chromosomes. Two transcription factors that may facilitate post-meiotic sex-linked gene expression are Heat Shock Transcription Factor 1 (HSF1), which localizes to sex chromatin^13^ and HSF2, which preferentially binds chromatin of Y-palindromic genes^14^. Consistent with this, the testis is known to have specialized transcription regulation strategies in post-meiotic testicular germ cells^15^ that appear to also apply to palindromic and multicopy X- and Y-linked genes.

This work leaves open the question as to why palindromes are heavily enriched on both the mammalian X and Y chromosomes. It is possible that X- and Y-chromosome palindromes are necessary for the long-term evolutionary stability of the genes they harbor in order to rapidly purge deleterious mutations via gene conversion^16^. Alternatively, the presence of two or more palindromic gene copies provides a larger substrate for new beneficial mutations to arise and become fixed, via gene conversion^17^. For both purging or fixing new mutations in palindrome arms, it will be important to assess arm-to-arm gene conversion rates.

## Materials and Methods

### Generation of 4930567H17Rik and Mageb5 palindrome arm inversions and deletions

To generate mice carrying palindrome arm inversions and deletions we used CRISPR with dual single guide RNAs (sgRNAs). sgRNAs were designed to unique sequences flanking the targeted palindrome arm, as close to the edge of the arm as possible (Supplementary Table 1). Since sgRNA cutting efficiency varies between sgRNAs, we tested their activity via pronuclear injection of an sgRNA/Cas9 expression plasmid, pSpCas9(BB)-2A-GFP (pX458)^18^ in mouse zygotes. The surviving mouse zygotes were allowed to develop into 64–128 cell blastocysts and the cutting efficiency of each sgRNA was determined by PCR of the cut sites (Supplementary Table 1) and Sanger sequencing of purified PCR products to identify edits at the target sites.

After active sgRNAs were identified for both sides of each targeted palindrome arm, two pX458M plasmids encoding the sgRNAs and Cas9 together with a single-stranded oligonucleotide were injected into the pronucleus of (C57BL/6JXSJL) F1 mouse zygotes. For *4930567H17Rik* and *Mageb5* palindrome arm inversions and for the *4930567H17Rik* palindrome arm deletion, we added single-stranded oligonucleotides with sequence homology flanking the junction boundaries, as listed in Supplementary Table 1, to promote the events. For the *Mageb5* palindrome arm deletion, we only used dual sgRNAs. The zygotes were generated from mating C57BL/6J females to (C57BL/6JXSJL) F1 males so that all targeted X chromosomes were C57BL/6J. Blastocysts from the pronuclear injection were implanted into pseudopregnant females. Genomic DNA from resulting pups was screened by PCR and Sanger sequencing of the inversion and deletion junctions. At least two independent mouse lines were obtained for inversions and deletions of the *4930567H17Rik* and *Mageb5* palindrome arms. Male and female mice were able to transmit both the *4930567H17Rik* and *Mageb5* deletions and inversions through the germline. Thus, their overall health was unaffected by CRISPR-mediated chromosome engineering.

### Mice and testis sample collection

Females heterozygous for an inversion or deletion of a single palindrome arm were repeatedly backcrossed to C57BL/6J. Backcrossing was performed up to seven generations, for two independent lines per genotype, to minimize any CRISPR-mediated off-target effects. The heterozygous females mating scheme resulted in wild-type and deletion/inversion male littermates which were directly compared in all experiments. Use of wild-type littermates as controls minimized the effects of genetic background and age. If wild-type littermates were not available, then we used age-matched controls from the same breeding line. To assess fecundity (average litter size per male), we mated mutant males to CD1 females. Testes were collected from 2–6 month old males for all experiments and weighed, along with total body, in order to determine relative testis weight. The alleles for the first mouse lines of each type were named *4930567H17Rik*^*InvArm1*^, *4930567H17Rik*^*DelArm1*^, *Mageb5*^*InvArm1*^, *Mageb5*^*DelArm1*^ with respective registered symbols In(X4930567H17Rik)1Jbmu, Del(X4930567H17Rik)1Jbmu, In(XMageb5)1Jbmu and Del(XMageb5)1Jbmu. All experiments performed on mice were approved by the University of Michigan Committee on Use and Care of Animals, and all experiments followed the National Institutes of Health Guidelines of the Care and Use of Experimental Animals.

### Preparation of adult testis cDNA and quantitative real-time PCR (qRT-PCR)

Intron-spanning primers (Supplementary Table 1), when possible, were used to perform qRT-PCR on adult testis cDNA preparations. One testis per mouse was used to isolate RNA using Trizol (Invitrogen) following the manufacturers recommendations. Five μg of total RNA was DNase treated with TurboDNase (Ambion) and reversed transcribed using Superscript II (Invitrogen) and oligonucleotide (dT) primers following the manufacturers protocol. qRT-PCR was performed in triplicate using Power SYBR Green master mix (Thermo Fisher Scientific) on a 7500 Real-time PCR thermalcycler (Applied Biosystems). Reactions were performed in 25 µl volumes, with 200 nM of each primer, 40 cycles of 95 °C for 15 seconds followed by 60 °C for 1 minute, and completed with a melt curve analysis. All PCR products produced a single peak based upon the melt curve analysis, indicating a single, specific, product is amplified in each reaction. We performed three technical replicates, per animal, for each qRT-PCR reaction. All comparisons of gene expression used the same standard reference control gene, *Trim42* (*Tripartite motif-containing 42*), because it is expressed specifically in the same post-meiotic testicular cells and at similar levels as *4930567H17Rik* and *Mageb5*. Normalized gene expression (∆*C*_T_) was calculated by subtracting *Trim42* crossing thresholds (*C*_T_) values from *4930567H17Rik* or *Mageb5 C*_T_ values, for each biological replicate. Control biological replicates (wild-type littermates from the same transgenic line) ∆*C*_T_ values were averaged. ∆∆*C*_T_ values were determined by subtracting the average ∆*C*_T_ of control samples from the ∆*C*_T_ values of mice carrying either a deletion or inversion. Fold changes in *4930567H17Rik* and *Mageb5* gene expression were determined via the 2^−∆∆CT^ method^19^. Statistical tests of differential gene expression were performed by comparing mean gene expression via unpaired, one-tailed, moderated t-tests.

### Testis Histology

Testes were fixed overnight in Bouin’s solution, paraffin embedded, sectioned to 5 μm, and stained with Periodic acid Schiff (PAS) and hematoxylin. Sections were visualized under a light microscope and specific germ cell populations were identified by their location within a tubule, nuclear size, and the nuclear staining pattern of chromatin^20^.

### FACs-based estimates of round spermatid frequencies

We largely followed a previously published protocol^21^ to isolate round spermatids (1n) and spermatocytes (4n). Briefly, we disassociated cells from a single testis by enzymatic treatment with Collagenase type I, DNase I (Worthington Biochemical Corporation), and Trypsin (Life Technologies). The cell suspension was passed through cell strainers (100 µm and 40 µm) and incubated with Hoechst 33342 (Life Technologies) to determine DNA content and propidium iodide (Acros Organics) to evaluate cell viability. Cell sorting was performed on a FACSAria II cell sorter (BD Biosciences). The purity of each sort was determined via fluorescence microscopy visual inspection of 100 cells morphology and nuclear staining with DAPI for round spermatids and with gH2AX antibody staining for pacyhtene spermatocytes. The purity of round spermatids was typically >85%, which is consistent with previous studies^21^.

### RNA-seq analyses

RNA-seq analyses were conducted by analyzing previously published datasets. Specifically, mouse tissue panel data were analyzed from SRP016501^22^, ovary data from SRP017959^23^, juvenile testis data from SRP018695^24^ and sorted testicular germ cell populations from SRP065082^25^ and SRP018124^26^. Alignments were performed using Tophat^27^ with the mm10 mouse reference genome, a refFlat file with RefSeq gene annotations and–max-multihits set to 240; otherwise standard default parameters were used. We used Cufflinks^27^, using the refFlat gene annotation file, to estimate expression levels as fragments per kilobase per millions of mapped fragments (FPKM).

### Dot plots

Self-symmetry triangular dot plots that show repeats within a sequenced region were generated from a custom Perl script that can be found at http://pagelab.wi.mit.edu/material-request.html.

### Data availability

All RNA-seq data analyzed in this study has been previously published and can be found in the NCBI short read archive (SRA).

## Electronic supplementary material

Supplementary Information
