# Supplementary material for: Male mice with large inversions or deletions of X-chromosome palindrome arms are fertile and express their associated genes during post-meiosis
Source: Sci Rep. 2018 Jun 12;8:8985. doi: 10.1038/s41598-018-27360-x (PMC5997626; doi:10.1038/s41598-018-27360-x)
Supplement: Supplementary file 1 — Supplementary Information [file 41598_2018_27360_MOESM1_ESM.pdf]

Male mice with large inversions or deletions of X-chromosome palindrome arms are fertile and express their associated genes during post-meiosis.

Supplementary Information

Alyssa N. Kruger, Quinn Ellison, Michele A. Brogley, Emma R. Gerlinger and Jacob L. Mueller

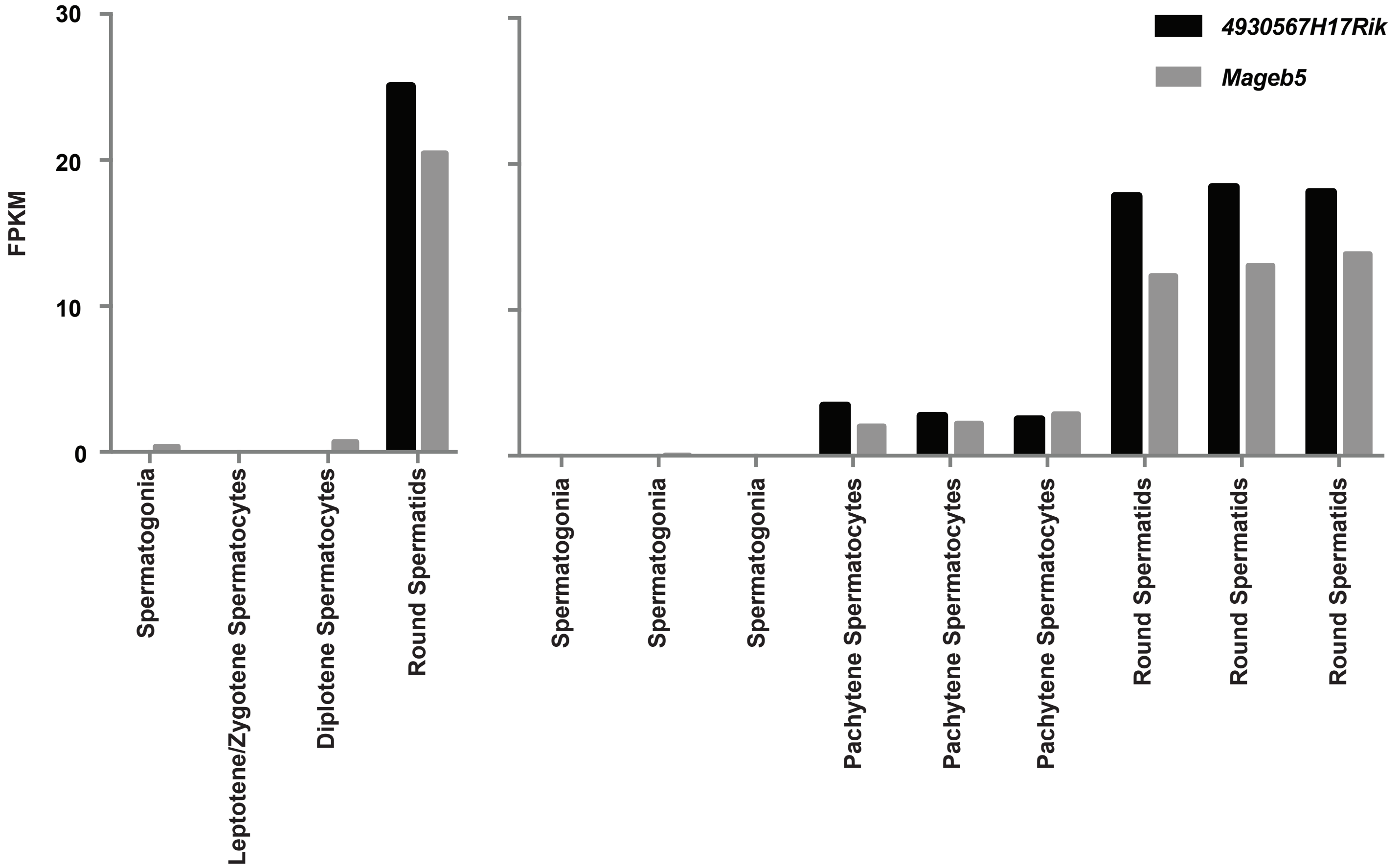

Supplementary Figure 1. Expression levels of 4930567H17Rik and Mageb5 genes in sorted spermatogenic populations from independent studies. Gene expression is shown as FPKMs (number of fragments per kilobase per million mapped fragments). The left panel shows data from Larson et al., 2016, Genetics, and the right panel shows three biological replicates from Soumillon et al., 2013, Cell Reports.

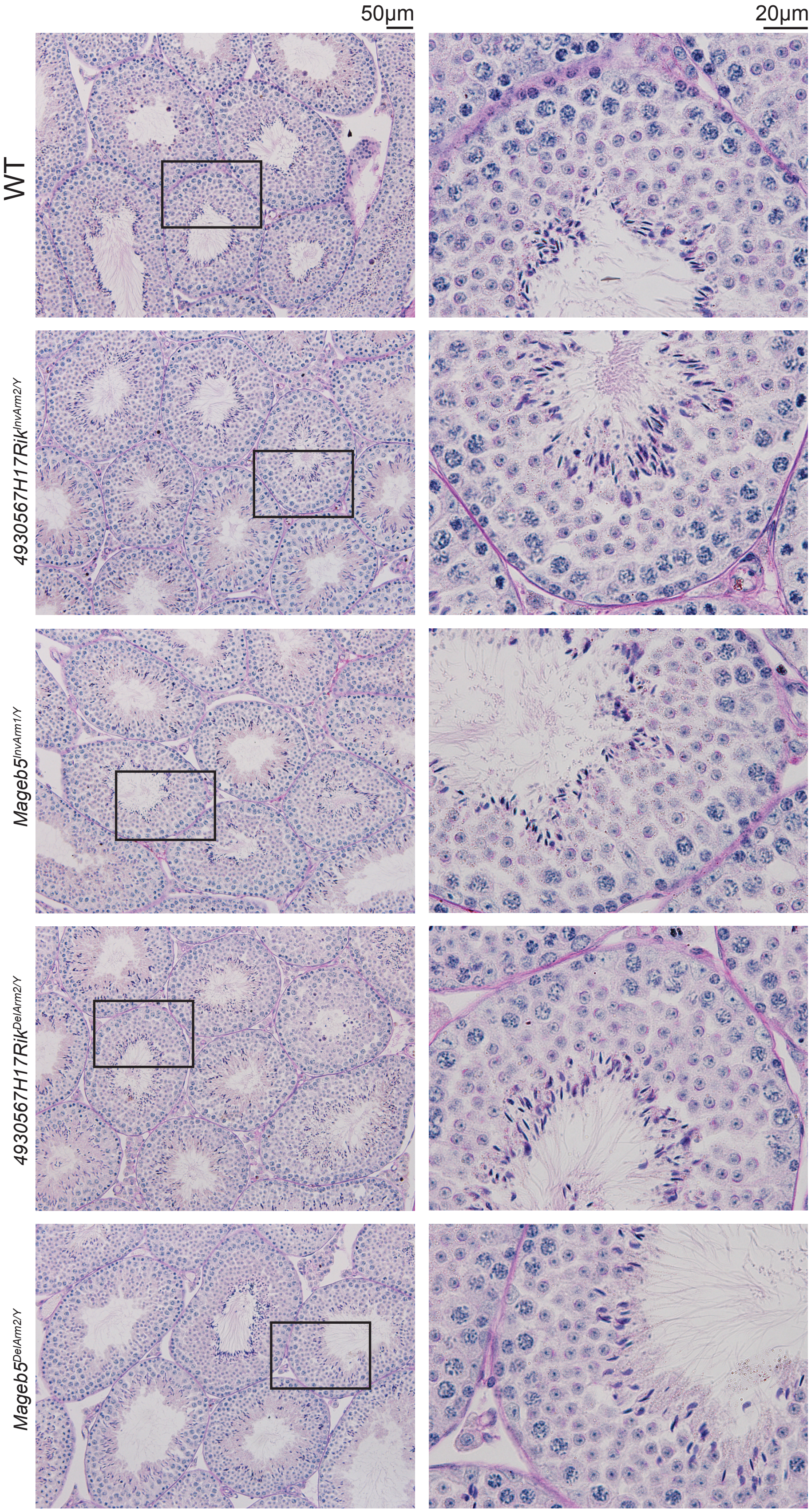

Supplementary Figure 2. Male mice carrying inversions or deletions of a single palindrome arm, for the 49305667H17Rik or Mageb5 X-palindromes do not exhibit detectable spermatogenic defects as based upon histological sections. Histological sections were stained with hematoxylin and periodic acid-Schiff (He-PAS). Low magnification images are shown to the left and higher magnification images of a representative, and boxed, seminiferous tubule are shown to the right. WT = wild-type.

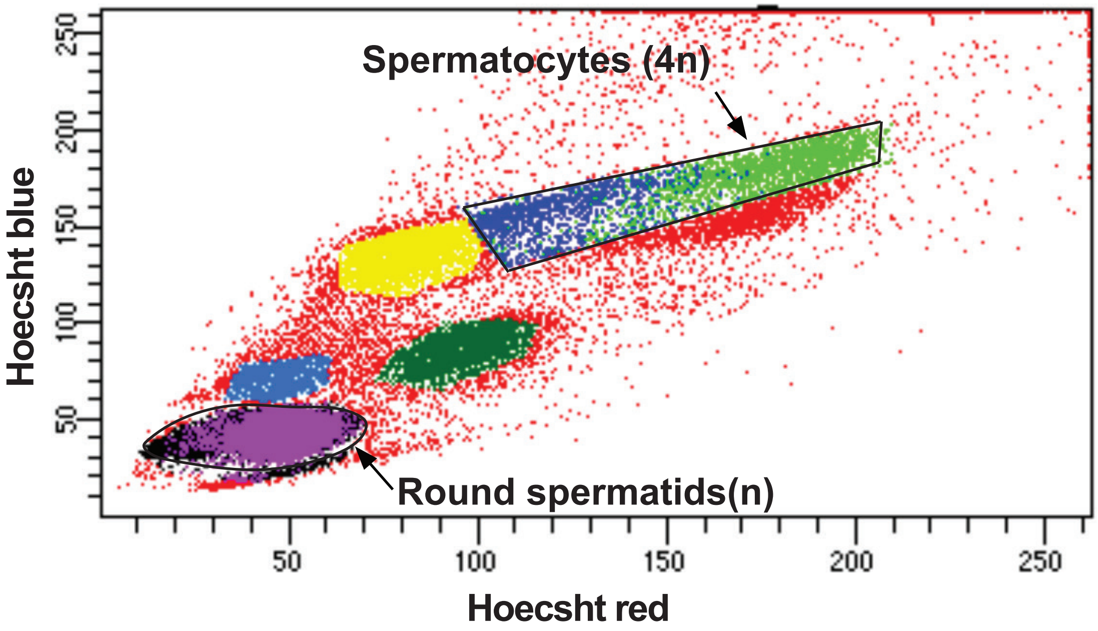

Supplementary Figure 3 Representative FACS plot of spermatogenic cell populations. Round spermatids (1n) and spermatocytes (4n) populations that were sorted are contained within the black outlines.

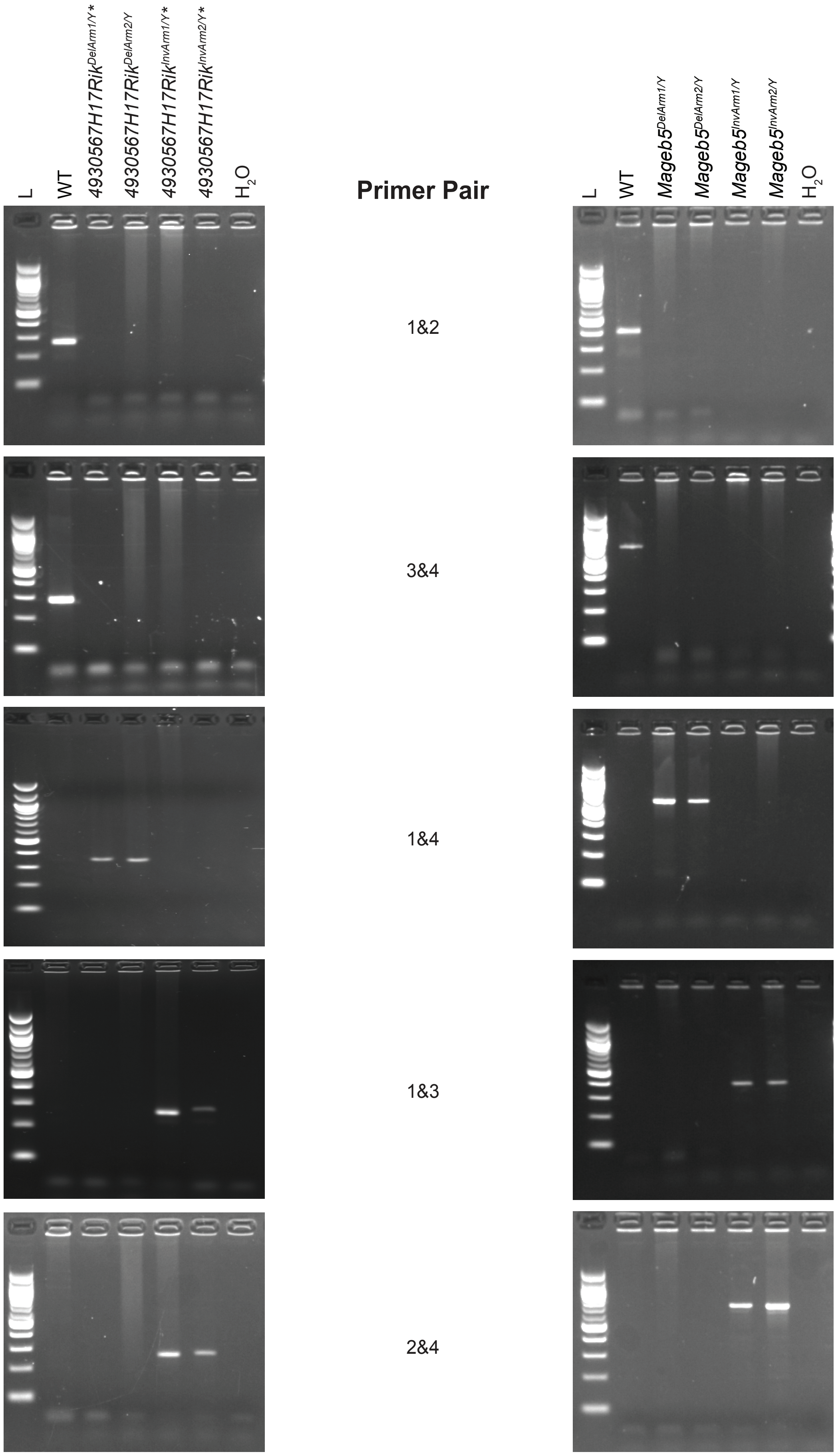

Supplementary Figure 4. Full-length agarose gels, with ladders (L), of PCR genotyping from Figure 2.

Supplementary Table 1. List of sgRNA sequences and oligos used in this study (5' to 3').

sgRNAs:

4930567H17Rik 5'	CCCAGACTTAGTGCATT
4930567H17Rik 3'	TGGTACGGGACTGACAA
Mageb5 5'	AAATACTCAACATGCAATGA
Mageb5 3'	AAAGTTTGATATCGCCTTGG

Single-stranded oligo donors:

*4930567H17Rik*_Deletion	AGTACGTGAACAGCATCCAGGGGCCCATGGGACAAGTCTGG GAGCAGTCACTGAGTTAACCAAGGGAGATTTCTCAGGCAACA CACCCCTATCCTAAGCGTTCTAAAGCATCCTAACCTT
*4930567H17Rik*_Inversion	CATGGGACAAGTCTGGGAGCAGTCACTGAGTTAACCCCAATC AAAGGAGGACATGCTGGATTACTTGGCATCTTTTATTGTGGTA CTTCCTCAGTCTGTACTCAGGATATTATCATTTCTACTAAGTGA ACCAGAGC
*Mageb5*_Inversion	TACCTTATTGGTATGTGTGAGTACAGCTGAGCCACTTCATTAT TTTAATGCTCTAAGTAGACAGAGTTTTCTGGGTATTCTGTTCTA GCACCATCATGGGGGCATTATATTCCCCTTCTTGCAGGAATCT AGTTATT

RT/ qRT-PCR primers:

4930567H17Rik F	GGGCCTCTGAGACCACAT
4930567H17Rik R	TCTGCATGGGTCGTATGA
Mageb5 F	GGGAGAATCATCCACTTCTGA
Mageb5 R	TGGATTTTCTTGGCAGGTTC
Trim42 F	GAAGCATCGTCACCTCCTCT
Trim42 R	CTTCTCGCATAGGCTGTGGT
Mageb5 SFV F	CCATGCAAACATTGCCTAGA
Mageb5 SFV R	CTCCCTGGGTACACTTGCAT

Genotyping Primers:

4930567H17Rik_1	TGCTGTTTTCTGGATTGCACA
4930567H17Rik_2	ACAGTTTTGGTTGTTGAGGGA
4930567H17Rik_3	ACGGCTCTGGTTCACTTAGT
4930567H17Rik_4	TAGGAACCACTTGCATGTCG
Mageb5_1	GTTTGCAGAGTTGTGGACTGATAC
Mageb5_2	ATCATTTTCCTTGTAGAGAACACAGC
Mageb5_3	TAAATACTAGTCTCTTCTCAAGATGTCC
Mageb5_4	TGCATTGATCAAAAGGGAGA
